# Transient Exposure of Enalapril Normalizes Prenatal Programming of Hypertension and Urinary Angiotensinogen Excretion

**DOI:** 10.1371/journal.pone.0146183

**Published:** 2015-12-31

**Authors:** Asifhusen Mansuri, Ayah Elmaghrabi, Susan K. Legan, Jyothsna Gattineni, Michel Baum

**Affiliations:** 1 Department of Pediatrics University of Texas Southwestern Medical Center, Dallas, Texas, United States of America; 2 Department of Internal Medicine University of Texas Southwestern Medical Center, Dallas, Texas, United States of America; The University of Manchester, UNITED KINGDOM

## Abstract

Maternal low protein diet programs offspring to develop hypertension as adults. Transient exposure to angiotensin converting enzyme inhibitors or angiotensin II receptor blockers can result in improvement in hypertension. Male rats whose mothers received a low protein diet during the last half of pregnancy were given either vehicle, continuous enalapril (CE) in their drinking water or were given transient enalapril exposure (TE) after weaning at 21 days of age. The TE group had enalapril in their drinking water for 21 days starting from day 21 of life. All rats were studied at 6 months of age. Vehicle treated rats whose mothers were fed a low protein diet were hypertensive, had albuminuria, and demonstrated upregulation of the intrarenal renin-angiotensin system as evidenced by higher urinary angiotensinogen and urinary angiotensin II levels. In low protein rats both continuous and transient exposure to enalapril normalized blood pressure, urinary angiotensinogen and urinary angiotensin II levels at 6 months of age, but only continuous administration of enalapril decreased urinary albumin excretion. These data support the importance of the intrarenal renin-angiotensin system in mediating hypertension in programmed rats and transient exposure to enalapril can reprogram the hypertension and dysregulation of the intrarenal renin-angiotensin system.

## Introduction

Several epidemiological studies have demonstrated that small for gestational age infants are at risk for developing hypertension in later life [1–6]. Animal models have been utilized to examine the pathogenesis of the hypertension in prenatal programming. Offspring of rats whose mothers were administered a low protein diet, prenatal glucocorticoids or surgically induced reduced uterine perfusion develop hypertension as adults [7–12]. The cause for the hypertension has remained enigmatic.

The renin-angiotensin system is a major regulator of blood pressure and various components of the systemic and intrarenal renin-angiotensin system have been found to be affected in programmed rats [9,11–15]. Consistent with the renin-angiotensin system playing a role in mediating the hypertension in programming, studies have shown that both captopril, an angiotensin converting enzyme inhibitor, and losartan, an angiotensin II receptor blocker, administered to the drinking water from 2–4 weeks resulted a reduction in blood pressure to levels comparable to the rats whose mothers were fed a normal protein diet [16,17]. Nifedipine, a calcium channel blocker did not affect blood pressure in programmed rats [16]. The antihypertensive effect of both captopril and losartan was still present at 12 weeks of age, 8 weeks after the drugs were discontinued [16,17]. Similarly, transient exposure to enalapril, an angiotensin converting enzyme inhibitor, from 3–6 weeks of age has been shown to reduce the blood pressure in offspring of mothers fed a low protein diet 10 weeks after the drug was discontinued [18]. The mechanism whereby transient administration of enalapril in programmed rats causes a sustained decrease in blood pressure is unknown. The purpose of this study was to examine whether the hypotensive effect of transient administration of enalapril is maintained into adulthood and to determine if there are components of the renin-angiotensin system that are reprogrammed by transient administration of enalapril that may explain how normalization of blood pressure is maintained despite discontinuing the angiotensin converting enzyme inhibition.

## Methods

### Animals

Pregnant Sprague Dawley rats were fed either a control diet containing 20% protein or an isocaloric 6% low protein diet from the 12^th^ day of gestation until birth. This is the same protocol used by our laboratory and others to study the effect of prenatal programming on hypertension [8,19–24]. We have previously shown that the number of neonatal rats born to mothers who were fed the two diets was not different (11.4 ± 0.6 pups in the control vs 13.0 ± 0.5 pups in the low protein group, p = ns) [24]. Neonates were weaned at 21 days and all rats were then placed on a control diet. Rats were studied as adults and were from at least four different litters in each group. Only males were studied as they are affected by prenatal programming more severely than females and to reduce variability of the results [25–28]. These studies were approved by the IACUC (T-2011-0151) of the University of Texas Southwestern Medical Center.

After weaning the rats were separated into six groups:

1. 20% Vehicle (20%)–The mother consumed a 20% protein diet while pregnant which was maintained while nursing and after birth. Four cc/L of ethanol was added to the drinking water of the offspring after weaning as the vehicle used to dissolve the enalapril from 3 weeks of age until time of study.
2. 6% Vehicle (6%)- The mother consumed a 6% protein diet during the last half of pregnancy. The mother was then fed a 20% protein diet after giving birth and while nursing, as were the offspring after weaning. Four cc/L of ethanol was added to the drinking water after weaning as the vehicle used to dissolve the enalapril from 3 weeks of age until time of study.
3. 20% Continuous Enalapril (20%CE)—After weaning the 20% protein fed rats were given enalapril (100 mg/L) in their drinking water continuously until the time of study [18].
4. 6% Continuous Enalapril (6%CE)—After weaning the 6% protein fed rats were treated with enalapril (100 mg/L) in their drinking water continuously until the time of study.
5. 20% Transient Enalapril (20%TE)—After weaning the 20% protein fed rats were given enalapril (100 mg/L) in their drinking water continuously for 21 days starting at 21 days of life and then placed on regular water containing ethanol vehicle.
6. 6% Transient Enalapril (6%TE)—After weaning the 6% protein fed rats were provided enalapril (100 mg/L) in their drinking water continuously for 21 days starting at 21 days of life and then placed on regular water containing ethanol vehicle.

### Measurement of Blood Pressure

Blood pressure was measured in the 6 groups at 6 months of age. The investigator that measured the blood pressure was blinded as to which group the rat belonged. Rats were trained for 4 days for measurement of systolic blood pressure. Briefly rats were placed in Lucite tubes and a cuff was inflated several times so that the rats become accustomed to the procedure. The blood pressure was measured using tail cuff with a CODA Blood Non-Invasive Pressure Analyzer (Kent Scientific Corporation, Torrington, CT), which uses a volume pressure recording. Blood pressures measured with this system correlate well with blood pressure measurements obtained by telemetry [29]. The average of six blood pressure readings was used as the blood pressure for that rat.

### Urine Collections

Rats were acclimatized to metabolic cages for three days with free access to food and water. Urine was then collected for 24 hours for measurement of creatinine, protein, albumin, angiotensinogen and angiotensin II. For the angiotensin II and angiotensinogen collection, the receptacle contained 50 μg pepstatin, 10 mg sodium azide, 300 nmol enalaprilat and, 125 μmol EDTA to prevent the degradation of angiotensinogen and angiotensin II as previously described [30–32]. The urine was stored at -80°C after collection.

### Blood collections

After measurement of blood pressure and the urine collection, blood was obtained by retro-orbital puncture. Rats were anesthetized with isoflurane and blood was collected with a heparinized capillary and placed in a chilled tube containing 20 μmol/l enalaprilat, 5 mmol/l EDTA, 10 μmol/l pepstatin A and 1 mmol/l 1,10-phenanthrolene (all chemicals were from Sigma Chemical Co., St. Louis, MO) to prevent peptide degradation [30–32]. The blood was centrifuged at 1,200 g for 10 min at 4°C and then stored at -80°C.

### Urine and Blood Assays

Urine protein was measured using the Bradford assay (Bio-Rad Laboratories, Hercules, CA). Urine albumin was assayed using a Nephrat rat urinary albumin enzyme immunoassay kit (Exocell, Philadelphia, PA). Capillary electrophoresis was used to measure urine and serum creatinine.

For measurement of blood and urine angiotensin II, we used a protocol similar to that previously described by our laboratory [30,33,34]. Briefly, samples (2cc of urine or 0.1 cc of plasma) were loaded on to phenyl cartridges (Phenomenex, Torrance, CA). The angiotensin II was eluted from the cartridges with methanol, which was then dried under a stream of nitrogen. The residue was dissolved with EIA buffer and angiotensin II measured using an Angiotensin II Enzyme Immunoassay Kit from SPI-Bio (Montigny le Bretonneux, France). Urinary angiotensin II was measured in picogram per ml and results were reported in both picogram per 24 hours and also normalized to urine creatinine. Urine and plasma angiotensinogen was assayed using an ELISA kit (Rat Total Angiotensinogen Assay Kit, Immuno-Biological Laboratories Co, Minneapolis, MN). Urinary angiotensinogen was measured in nanogram per ml and results were reported in both nanogram per 24 hours and angiotensinogen (ng) to creatinine (mg).

Plasma renin activity was measured with a Gamma Coat Plasma Renin Activity ^125^I RIA kit (DiaSorin Inc., Stillwater, MN) by assaying angiotensin I generation. Angiotensin I was then measured using a radioimmunoassay. Serum aldosterone was measured using an ELISA kit (Enzo Life Sciences, Farmingdale NY) as per the manufacturer’s instructions.

### Statistical Analysis

Data are shown as the mean ± the standard error of the mean. Comparisons between the groups were assessed using analysis of variance with a post hoc Student-Newman-Keuls test.

## Results

**Fig 1:**
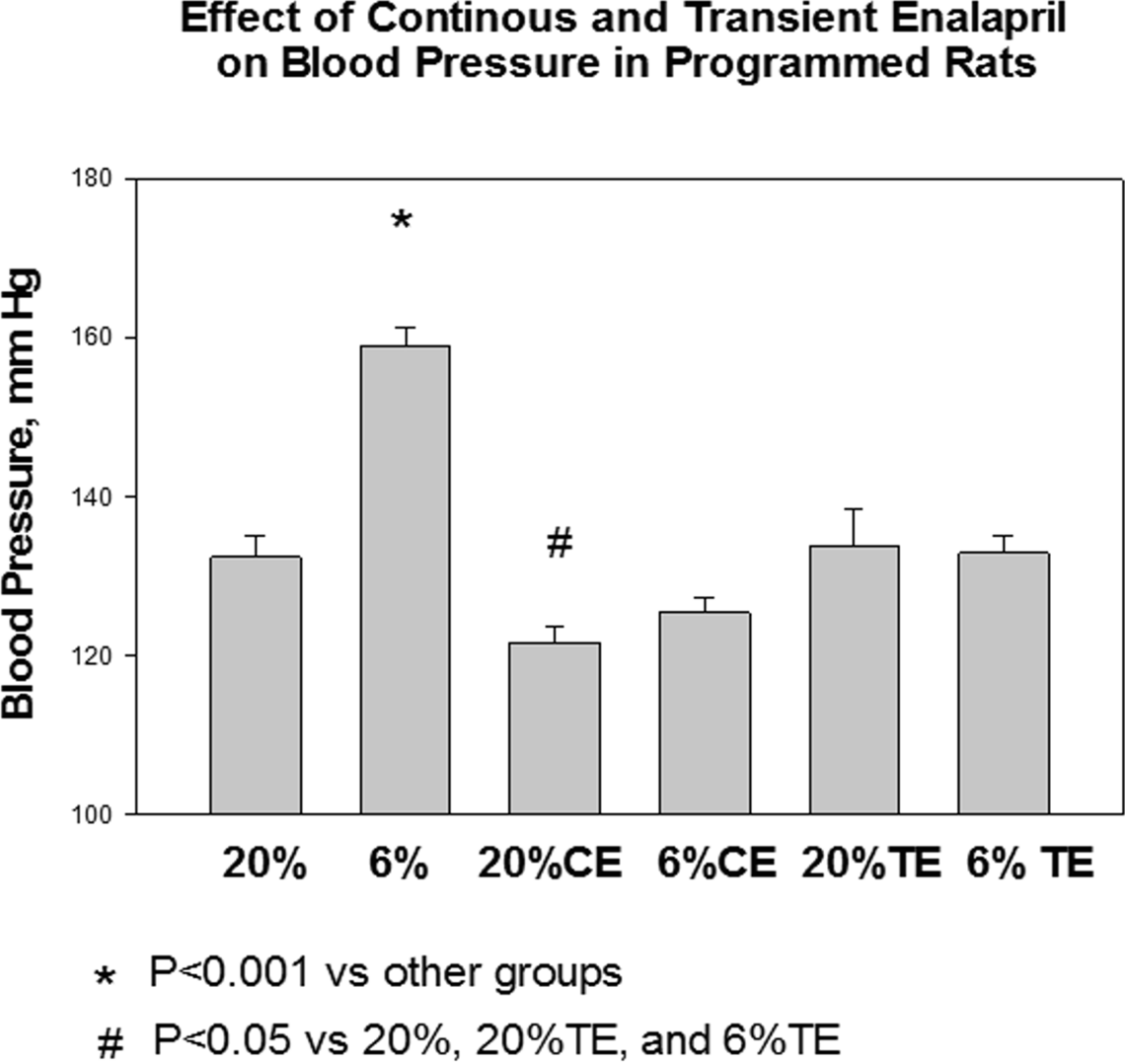
Effect of continuous and transient enalapril on blood pressure in control and programmed rats. Pregnant rats were given either a 20% protein diet or an isocaloric low protein diet during the last half of pregnancy. Nursing mothers were all given a 20% protein diet. Upon weaning at 3 weeks of age, rates were given enalapril (100 mg/L) or vehicle (ethanol 4 ml/L) in their drinking water. The continuous enalapril group (CE) received enalapril continuously until the time of study at 6 months of age. The transient enalapril group (TE) received enalapril from 3–6 weeks of age and vehicle thereafter. Blood pressure was measured by tail cuff after 4 days of training. The blood pressures were measured in a blinded fashion. The 6% group had a higher blood pressure than all the other groups. The continuous enalapril groups had a lower blood pressure than the corresponding transient enalapril group. N = 9–12 in each group.

In the first series of experiments we measured blood pressure in the 6 groups in a blinded fashion as shown in Fig 1. The blood pressure of the 6% vehicle group was higher than the 20% vehicle group as we have shown previously [8,19,20]. The 6% group that received continuous enalapril (6%CE) had a reduction in blood pressure to a value not different than the 20% group and the 20%CE group. Interestingly, the 6% transient enalapril group (6%TE) had a blood pressure comparable to the 20% and 20% TE groups despite the fact that they had not received enalapril for 20 weeks. The 20% continuous enalapril group had a lower blood pressure than the 20% group and the 6% and 20% transient enalapril groups.

To assess whether the hypertension in programming was mediated by dysregulation of the systemic renin-angiotensin system or if the systemic renin-angiotensin system was affected by enalapril, we measured plasma renin activity, angiotensin II and aldosterone in the 6 groups. As shown in Table 1, there was no difference in plasma angiotensinogen, plasma renin activity, angiotensin II and aldosterone levels in the programmed compared to the control rats. Continuous enalapril increased the renin level of both the 6% and 20% group (P<0.01), but neither transient nor continuous enalapril affected the levels of angiotensinogen, angiotensin II or aldosterone in any group.

**Table 1:** Effect of Continuous or Transient Enalapril on Systemic Renin-Angiotensin System in Control and Programmed Rats.

	Angiotensinogen (ng/ml)	Renin (ng/ml/hr)	Angiotensin II (pg/ml)	Aldosterone (pg/ml)
20%	8732 ± 302	2.7 ± 0.7	40.3 ± 8.2	224 ± 26
6%	7953 ± 704	2.7 ± 1.0	60.0 ± 19.5	268 ± 29
20%CE	7356 ± 516	31.4 ± 8.6	46.7 ± 7.0	310 ± 24
6%CE	8558 ± 462	34.8 ± 7.2	26.5 ± 5.7	285 ± 32
20%TE	8076 ± 665	1.6 ± 0.3	27.1 ± 5.9	373 ± 115
6%TE	7620 ± 489	2.0 ± 0.4	28.7 ± 9.7	204 ± 25

N = 9–15 in each group

The weights of the rats are shown in Table 2. The 6% groups weighed less than the 20% groups that were given the same postnatal treatment with vehicle, TE or CE (P<0.01). The continuous enalapril groups had less weight gain than the respective vehicle and TE groups (P<0.05). There was no difference in weight between the 6% group and the 20% CE group. The 20% CE group had a comparable weight to the 6% and 6% TE group and weighed less than the 20% TE and 20% group. This reduction in body weight was not seen in transient enalapril groups. The 20% group had comparable weight to the 20% TE group and the 6% group had a comparable weight to the 6% TE group.

**Table 2:** Effect of Continuous or Transient Enalapril on Creatinine Clearance and Urinary Protein Excretion in Control and Programmed Rats.

	Body Weight gm	Creatinine Clearance (ml/min)	Creatinine Clearance/100gm BW	U Protein Excretion (mg/24h)	U Protein Excretion (mg/24hour/100gm BW)	U Albumin Excretion (mg/24h)	U Albumin Excretion (mg/24hour/100gm BW)
20%	502 ± 19	2.72 ± 0.15	0.54 ± 0.03	57.8 ± 6.7	11.85 ± 1.3	5.7 ± 1.0	1. 1 ± 0.2
6%	412 ± 10	2.03 ± 0.21	0.49 ± 0.05	69.3 ± 16.0	16.7 ± 3.8	24.9 ± 7.3	6.0 ± 1.8
20%CE	442 ± 5	2.44 ± 0.12	0.55 ± 0.03	20.1 ± 4.1	4.5 ± 0.9	1.4 ± 0.9	0.3 ± 0.2
6%CE	375 ± 10	2.11 ± 0.11	0.56 ± 0.02	25.2 ± 2.8	6.7 ± 0.6	4.9 ± 2.8	1.3 ± 0.6
20%TE	494 ± 21	2.78 ± 0.29	0.56 ± 0.06	44.0 ± 5.8	8.9 ± 1.2	10.2 ± 2.8	2.1 ± 0.6
6%TE	428 ± 11	2.60 ± 0.15	0.61 ± 0.03	51.8 ± 8.7	11.9 ± 7.8	19.2 ± 5.9	4.4 ± 1.4

N = 9–15 in each group

We measured creatinine clearance in rats at 6 months of age. The results are shown in Table 2. The uncorrected creatinine clearance was lower in the 6% group compared to the 20% group using an unpaired t test (p<0.05), however when all groups were compared using analysis of variance there was no difference between the groups. Importantly, when the creatinine clearance was corrected for body weight, the values in all the groups were quite comparable. Thus, prenatal programming did not affect creatinine clearance at 6 months of age when the results were normalized for body weight and enalapril did not affect creatinine clearance in any group.

Urinary protein and albumin excretion in the 6 groups are shown in the Table 2. Urine protein excretion per 24 hours and per 24 hours normalized for body weight was not affected by prenatal programming. Continuous administration of enalapril resulted in a decrease in urinary protein excretion in both the 20% and the 6% protein groups (P<0.05). There was a small reduction in protein excretion in the 20%TE group to a level less than the 6% group (P<0.05). However, urinary albumin excretion per 24 hours and per 24 hours normalized per body weight was greater in the 6% group compared to the 20% group (P<0.05). Continuous administration of enalapril resulted in a reduction in albumin excretion in the 6% group (6%CE) to a level less than the 6% group not treated with enalapril and the 6% TE group (P<0.05). Transient administration of enalapril did not result in a significant reduction in urinary albumin excretion in programmed rats.

**Fig 2:**
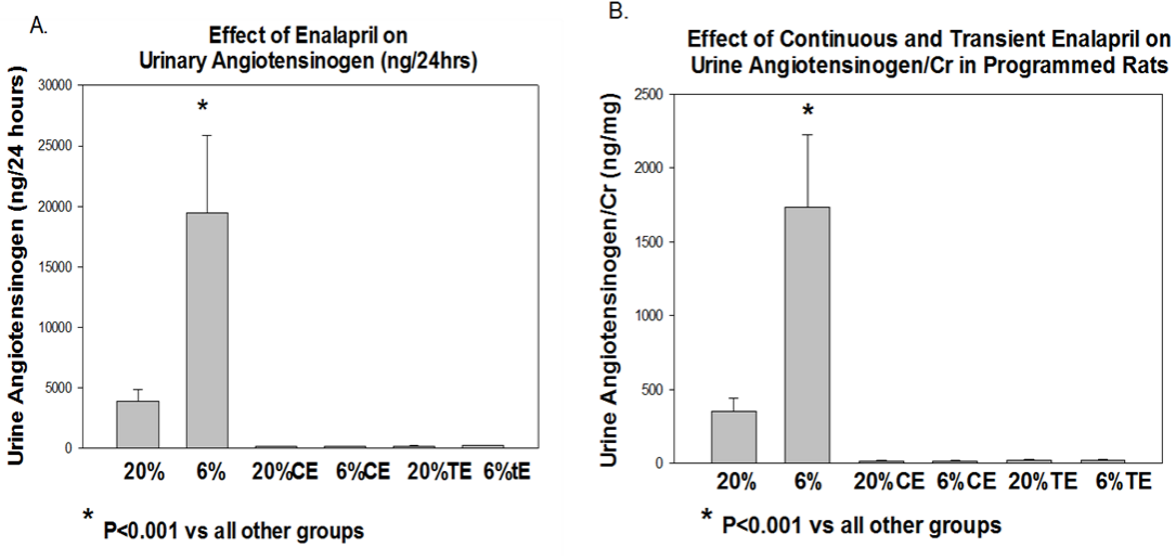
Effect of continuous and transient enalapril on urine angiotensinogen levels in control and programmed rats. Rats from the 6 groups were placed in metabolic cages at 6 months of age. 24 hour total urine angiotensinogen (A) and urinary angiotensinogen/creatinine (Cr) levels (B) were measured in control and prenatal programmed rats. Urine angiotensinogen /24 hours and urinary angiotensinogen/cr were higher in the offspring of the 6% rats compared to all other groups. N = 11–16 in each group.

Urinary angiotensinogen has been shown to be a marker of the activity of the intrarenal renin angiotensin system [31,32,35]. In Fig 2, we show that urinary angiotensinogen is elevated in the 6% group compared to the 20% group as well as the continuous and transient enalapril groups whether normalized to the 24 hour urine volume (A) or urine creatinine concentration (B). Thus, the intrarenal renin-angiotensin system is increased in the programmed offspring which is normalized by continuous and transient treatment with enalapril. It should be noted that when comparing the 6 groups in Fig 2, there was no statistical differences in the urinary angiotensinogen in the 20% vehicle group compared to the 20% TE and 20% CE groups. However, if one compared the effect of enalapril on the 20% groups independent of the 6% groups, continuous and transient enalapril did decrease the urinary angiotensinogen excretion compared to the 20% vehicle group (p<0.001).

**Fig 3:**
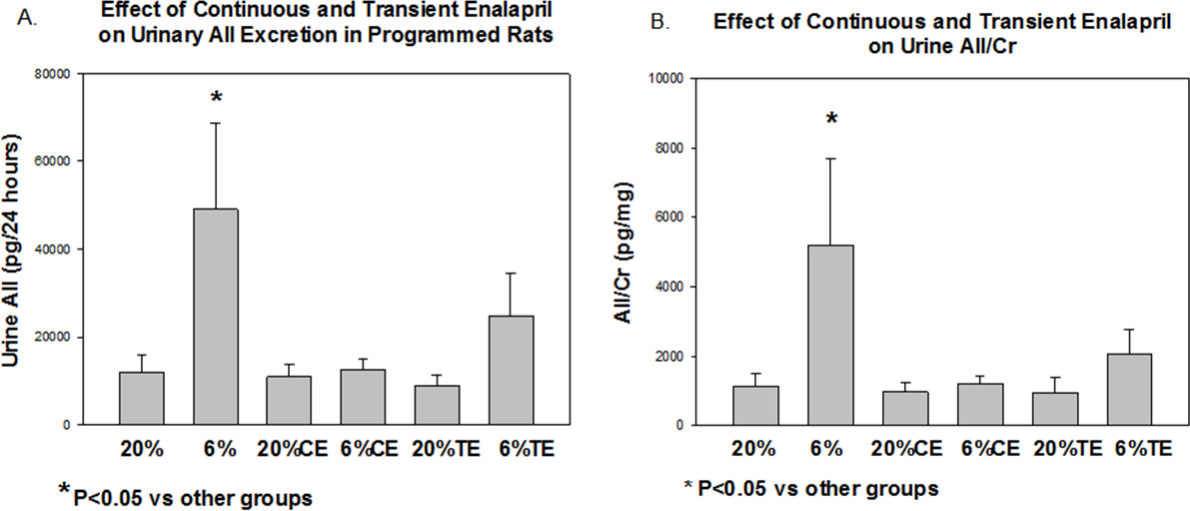
Effect of continuous and transient enalapril on urine angiotensin II levels in control and programmed rats. Rats from the 6 groups were placed in metabolic cages at 6 months of age. 24 hour total urine angiotensin II (A) and urinary angiotensin II/creatinine (Cr) levels (B) were measured in control and prenatal programmed rats. Urine angiotensin II/24 hours and urinary angiotensin II/Cr were higher in the offspring of the 6% rats compared to all other groups. N = 11–16 in each group.

We next examined if prenatal programming by maternal dietary protein restriction affected urinary excretion of angiotensin II and if urinary angiotensin II was affected by transient or continuous administration of enalapril. As shown in Fig 3, prenatal programming resulted in a significant increase in urinary angiotensin II excretion compared to the 20% control when normalized per 24 hour urine volume (A) or when normalized per urinary creatinine (B). Of importance, both continuous and transient administration of enalapril to the rats whose mothers were fed a low protein diet caused a significant reduction in urinary angiotensin II excretion.

## Discussion

The present study examined whether the prenatal programming of hypertension due to maternal dietary protein deprivation was affected by enalapril. We find that both continuous and transient therapy with enalapril normalized the blood pressure in adult offspring due to maternal dietary protein deprivation. The importance of this study is that the transient administration of enalapril can reprogram the hypertension seen with prenatal programming. The normalization of blood pressure in programmed rats had previously been demonstrated for 8–10 weeks after discontinuing either an angiotensin converting enzyme inhibitor or an angiotensin II receptor blocker [16–18]. In the present study the blood pressures were examined 20 weeks after discontinuing enalapril in the transient enalapril groups showing that the effect of transient enalapril remains well into adulthood.

We examined the effect of prenatal programming in offspring of mothers that were fed a low protein diet on creatinine clearance, protein and albumin excretion. While there was no effect of prenatal programming on urinary protein excretion in this study examining rats at 6 months of age, the programmed rats had a significantly greater urinary albumin excretion compared to the 20% controls. The decrease in creatinine clearance in the 6% group did not reach statistical significance compared to the other groups. We had previously examined if prenatal programming affected urinary protein and albumin excretion in offspring of mothers fed a low protein diet compared to those fed a normal protein diet. When studied at a year and a half of age we found that there was no effect of prenatal programming on either urinary protein or albumin excretion despite the fact that there was a significant decrease in glomerular filtration rate in the programmed rats [20]. With the progressive decline in glomerular filtration rate in programmed rats [20], it is not surprising that there is an increase in urinary albumin excretion in the current study. The reason for the lack of albuminuria in our previous study examining older rats is unclear. In this study we find that continuous but not transient enalapril treatment decreased albumin and protein excretion, which is consistent with the effect of angiotensin converting enzyme inhibitors to decrease glomerular capillary pressure [36].

Previous studies have examined the importance of the systemic renin-angiotensin system in the programming of hypertension [13]. There is some variability in how programming affects the systemic renin-angiotensin system, which is dependent on the nature of the prenatal insult and the time after birth when the renin-angiotensin system is assessed. Studies examining the effect of a maternal low protein diet have shown that plasma renin activity is lower at 4 weeks in programmed rats compared to controls, with a smaller but still significant reduction in plasma renin activity at 8 weeks of age [37]. This same group found higher levels of plasma renin activity in the low protein group compared to controls when studied at 16 weeks and 11 months of age [18,21]. Transient administration of enalapril normalized the plasma renin activity at 16 weeks in programmed rats [18]. Others have found no difference in plasma renin activity at 13 weeks in offspring of rats fed a low protein diet compared to a normal protein diet [38]. Comparable plasma angiotensin II levels are measured in programmed and control rats at 4 weeks of age and 13 weeks of age [38,39]. Aldosterone levels are higher in rats whose mothers were fed a low protein diet at 4 weeks of age compared to controls and a smaller but significant increase was found at 8 weeks of age [37]. We have found a higher serum aldosterone level in rats whose mothers were fed a low protein diet at 4 months of age compared to controls and a corresponding increase in cortical collecting duct sodium transport in programmed rats [40]. Others have found a comparable aldosterone level at 16 weeks in programmed rats on a normal salt diet but a significantly higher level than control when the rats were placed on a high salt diet [18]. In the present study, we found no effect of maternal low protein diet on the offspring at 6 months of age and neither transient nor continuous enalapril affected the systemic angiotensin II or aldosterone levels. Plasma renin activity was comparably elevated in both the 6% and 20% group that received continuous but not transient enalapril. Consistent with our results, others have found that transient enalapril did not affect the levels of aldosterone in programmed rats [18].

Kobori et al. showed that urinary angiotensinogen is an indirect indicator of the intrarenal renin-angiotensin system and that urinary angiotensinogen levels correlate with intrarenal angiotensin II levels but not with plasma angiotensin II levels [31,32]. Urinary angiotensinogen and intrarenal angiotensin II increased with angiotensin II mediated hypertension but not in rats with hypertension treated with deoxycorticosterone acetate/high salt diet [31].

Previous studies have examined the effect of prenatal programming on the intrarenal renin-angiotensin system. Offspring of rats whose mothers were fed a low protein diet had comparable renal angiotensin I and angiotensin II levels at 28 days of age to offspring of rats fed a control diet [39]. Prenatal administration of dexamethasone programs offspring to develop hypertension [25,41]. There was a higher concentration of urinary angiotensin II/creatinine in programmed rats compared to controls [30]. Offspring of rats that were of low birth weight due to surgical reduction in uterine perfusion had reduced renal renin and angiotensinogen mRNA expression compared to controls at 1 day of age, but the programmed rats had significantly higher levels of both at 16 weeks of age [42]. There was no difference renal ACE mRNA or protein abundance at 16 weeks but the ACE activity was higher in programmed than control offspring [42]. Despite the evidence for increased renal angiotensin II production in the latter two studies, there was no demonstrable difference in renal angiotensin II content [30,42]. The reason for this discrepancy is not clear.

Recent studies had examined urinary angiotensinogen in neonates and as a marker of activation of the intrarenal renin angiotensin system in programmed rats. Urinary angiotensinogen/creatinine ratio was found to be higher in preterm than in full term neonates [43,44] and may serve as a marker for acute kidney injury [43]. Interestingly a study examined the effect of prenatal programming using a model of uterine artery ligation. At 32 weeks of age, programmed rats had no difference in plasma angiotensinogen compared to control rats, while, compared to controls, the programmed rats had significantly higher urinary angiotensinogen/creatinine levels at 20 and 32 weeks of age [45]. These findings are in concordance with our findings showing that prenatal programming has no effect on plasma angiotensinogen levels but causes a marked increase in urinary excretion of angiotensinogen.

In all of previous studies and the current study, the angiotensin converting enzyme inhibitor or angiotensin II receptor blocker was given to young animals at or near the time of weaning [16–18]. The effect of transient administration of angiotensin converting enzyme inhibitors and angiotensin II receptor blockers on blood pressure lasted for weeks after the drug has been discontinued. However, it is unclear if there is a window where this reprogramming of blood pressure can occur. It is unclear if transient administration of enalapril would continue to have an effect if administered to a programmed adult and how long the effect would last. It is also unknown if the effect of an angiotensin converting enzyme inhibitor or angiotensin II receptor blocker will have a comparable effect in humans who are at risk for hypertension because of being small for gestational age or very premature. Clearly, one cannot administer angiotensin converting enzyme inhibitor or angiotensin II receptor blockers during nephrogenesis in very premature infants because of the importance of the renin angiotensin system in renal development [46,47]. Whether one could administer an angiotensin converting enzyme inhibitor or angiotensin II receptor blocker to humans to prevent hypertension at a time after renal development has occurred and for how long is unknown but of clinical importance. It should be noted that continuous administration of enalapril caused the rats to have a lower weight than the vehicle treated and the transient enalapril group. Whether this would occur in humans is unknown.

In summary, we demonstrate that there was a significant decrease in both urinary angiotensinogen and angiotensin II by continuous and transient administration of enalapril. These data are consistent with the intrarenal renin-angiotensin system playing a role in mediating the hypertension with prenatal programming. The fact that transient administration of enalapril can normalize blood pressure and urinary angiotensin II and urinary angiotensinogen levels suggest that there may be a window during postnatal development when prenatal programming can be reprogrammed.
